# Oral and maxillofacial lesions in older individuals and associated factors: A retrospective analysis of cases retrieved in two different services

**DOI:** 10.4317/jced.56194

**Published:** 2019-10-01

**Authors:** Mª Fernanda-Lopes Fonseca, Camila-de-Nazaré-Alves-de Oliveira Kato, Mateus-José-de Carvalho Pereira, Lucas-Tadeu-Ferreira Gomes, Lucas-Guimarães Abreu, Felipe-Paiva Fonseca, Ricardo-Alves Mesquita

**Affiliations:** 1 DDS, MSc, Department of Oral Pathology and Surgery, School of Dentistry, Universidade Federal de Minas Gerais, Belo Horizonte, MG, Brazil; 2 DDS, MSc, PhD Student, Department of Oral Pathology and Surgery, School of Dentistry, Universidade Federal de Minas Gerais, Belo Horizonte, MG, Brazil; 3 Undergraduate Student, School of Dentistry, Universidade Federal de Minas Gerais, Belo Horizonte, MG, Brazil; 4 DDS, MScPhD, Adjunt Professor, Department of Paediatric Dentistry and Orthodontics, School of Dentistry, Universidade Federal de Minas Gerais, Belo Horizonte, MG, Brazil; 5 DDS, MSc, PhD, Adjunt Professor, Department of Oral Pathology and Surgery, School of Dentistry, Universidade Federal de Minas Gerais, Belo Horizonte, MG, Brazil; 6 DDS, MSc, PhD, Full Professor, Department of Oral Pathology and Surgery, School of Dentistry, Universidade Federal de Minas Gerais, Belo Horizonte, MG, Brazil

## Abstract

**Background:**

Studies on the oral and maxillofacial lesions (OMLs) in older people usually assess data of laboratory services and data from oral medicine clinic have been poorly described. The aim of this study was to describe and to compare OMLs in older individuals considering two data sources, besides to assess associated factors with the three most frequent lesions.

**Material and Methods:**

A retrospective study was conducted with individuals aged 60 years or older. Data of individuals and lesions reported in both services were collected. Univariate analysis was used to test the association between the occurrence of the lesion and the independent variables. The level of significance was set at 5%.

**Results:**

A total of 1,695 (37.3%) records were from the Oral Medicine clinic and 2,848 (62.7%) from the Laboratory service. Inflammatory/reactive lesion group was the most frequent in both services (40.4% in Oral Medicine Clinic and in 44.2% Laboratory). The second and third groups of lesions in the Oral Medicine clinic were infectious diseases (18.5%), and variations of normality (10.8%), while in the laboratorial service were the malignant neoplasms (17.6%) and potentially malignant disorders (13.3%). Differences between services regarding the frequency of lesion groups occurred (*p*<0.05), except for pigmented (*p*=0.054) and infectious (*p*=0.054) groups. Females (OR: 2.08; CI: 1.81–2.39) and individuals who wore a removable prosthesis (OR: 3.99; CI: 2.83–5.62) were also likely to have inflammatory fibrous hyperplasia. Old–old individuals (OR: 1.70; CI: 1.30–2.21), male (OR: 3.63; CI: 3.00–4.39), smoking (OR: 6.05; CI: 4.84–7.56) or alcohol use (OR: 3.95; CI: 3.12–5.01) were likely to have squamous cell carcinoma.

**Conclusions:**

The results showed different frequencies of OMLs in older individuals according to the data sources and age group. The findings are important to direct public policies for this age group.

** Key words:**Oral medicine, laboratory service, older adult, oral diagnosis, oral and maxillofacial pathology.

## Introduction

The World Health Organization (WHO) has reported changes in demographic trends, in particular for the older population. The number of individuals over 65 years of age was 524 million in 2010. Over the next 40 years, this number is expected to skyrocket to more than 1.5 billion. In developing countries, the increase in the number of older individuals may reach 250%, while in developed countries, this figure may be 71% (1). The estimate of the number of older individuals in Brazil is quite similar to the global projections. According to the Brazilian Institute of Geography and Statistics, in 2010, the number of individuals aged 60 years or more was 19.6 million. This number will soar to 66.5 million in 2050 (2).

During the course of aging, an individual might become vulnerable to certain oral diseases. This vulnerability is associated with the thinning of the oral epithelium due to salivary changes, fat loss, reduced cell proliferation, and degeneration of collagen fibers, which will consequently impair the immune response to stimuli (3). Oral and maxillofacial pathologists have been concerned in determining the most frequent oral diseases affecting older individuals. For this purpose, epidemiological studies aiming to assess the prevalence or frequency of oral and maxillofacial lesions (OMLs) have been conducted worldwide (4-6).

Prevalence studies evaluating samples of individuals to determine the frequency of a disease have demonstrated that the most prevalent OML among older people are denture-induced stomatitis (7), Fordyce granules (4), fissured tongue, and sublingual varicosities (8,9). However, retrospective studies assessing data from services have showed that, usually, old individuals are affected by at least one lesion. When the frequency of the OMLs was computed, the types of lesions most commonly observed among older people included inflammatory/reactive lesions, malignant neoplasms (10,11), infectious diseases, and potentially malignant disorders (5).

Retrospective studies on the OMLs observed among older individuals usually involve the assessment of data of biopsied specimens determined by laboratory services (10-13). However, data on OMLs that do not require laboratory analysis for diagnosis confirmation have been under-documented, leading to inaccurate information about the frequency of OMLs. Therefore, the main objective of this study was to describe and compare the frequency of OMLs in individuals aged 60 years or older considering two different data sources: an Oral Medicine clinic and a laboratory service. The present study also assessed the association of the three most frequent lesions with demographic and oral behavior variables.

## Material and Methods

-Study Design and Ethical Issues

A retrospective cross-sectional study was performed using data obtained from Oral and Maxillofacial Pathology services. The study was undertaken in accordance with the Declaration of Helsinki and was approved by the Ethics Committee of the Federal University of Minas Gerais (protocol number: 10723019.0.1001.5149).

-Sampling and Setting

Data were obtained from the Oral Medicine clinic and the laboratory service of the Division of Oral and Maxillofacial Pathology at the Department of Oral Pathology and Surgery of the Federal University of Minas Gerais, Belo Horizonte, Minas Gerais, Brazil. Oral Medicine clinic data from 1990 to 2017 and laboratory data from 2001 to 2017 were reviewed. In our department, patients’ evaluation, treatments and diagnoses performed in the Oral Medicine clinic have been conducted by a team of specialists in Oral Medicine. When necessary, biopsies are performed and evaluated by an oral and maxillofacial pathologist.

All records included in the study belonged to individuals aged 60 years or older. For the purposes of the present analysis, the sample was divided into two groups according to the guidelines of the National Institute of Applied Economic Research: young–old individuals (60–79 years) and old–old individuals (80 years or older) (14). Records with incomplete data or records with inconclusive diagnosis were excluded. In the case of duplicate records (the same patient with records in the Oral Medicine clinic and in the laboratory service), only the record from the Oral Medicine clinic was computed.

When histopathological analysis was necessary (for instance, individuals with squamous cell carcinoma, odontogenic cysts, odontogenic tumors, fibrous hyperplasia, leukoplakia or other lesions), patients underwent biopsy. Individuals with oral and maxillofacial lesions, for which histopathologic analysis (for instance Fordyce’s granules, varices, florid cemento-osseous dysplasia or others) was not necessary, did not undergo biopsy.

Data from the laboratory service, whose histopathological findings were not accompanied by enough clinical information for the final diagnosis were excluded. For instance, hyperkeratosis was considered a non-conclusive histopathological diagnosis and, therefore, was excluded. However, cases of hyperkeratosis with a degree of epithelial dysplasia and cases with clinical diagnosis of leukoplakia were included in the lesion group of potentially malignant disorders. During data collection, the term leukoplakia was used for epithelial dysplasia and leukoplakia.

-Data Collection

Data on participants’ age, sex, and self-reported skin color, as well as information on behaviors, such as smoking, use of alcohol, and removable prosthesis wearing were obtained. In addition, data on the lesions, including symptoms, anatomical location, and lesion group were evaluated. Benign and malignant neoplasms were classified according to the 2017 WHO classification (15). The classification of other lesions was based on the categories described in the Textbook of Oral and Maxillofacial Pathology (16). In this study, the lesions were assigned to groups following a similar grouping strategy used in previous studies of this research group (13,17). Information on variations of normality was also collected.

-Statistical Analyses

Data were analyzed using the Statistical Package for the Social Sciences (SPSS) software (version 20.0; IBM Corp., Armonk, NY, USA). Descriptive analyses were carried out. Differences between the two services regarding the frequency of lesions and group of lesions were evaluated with the Pearson chi-square test. After data of the two services were pooled, the association of the groups of lesions with the age groups was assessed.

Finally, univariate analyses were carried out to test the association between the occurrence of the three most frequent lesions and the independent variables age, sex, skin color, smoking, use of alcohol, and removable prosthesis wearing. The results were provided in terms of odds ratio (OR), confidence interval (CI), and *p* values. The level of significance was *p*<0.05.

## Results

**Figure 1:**
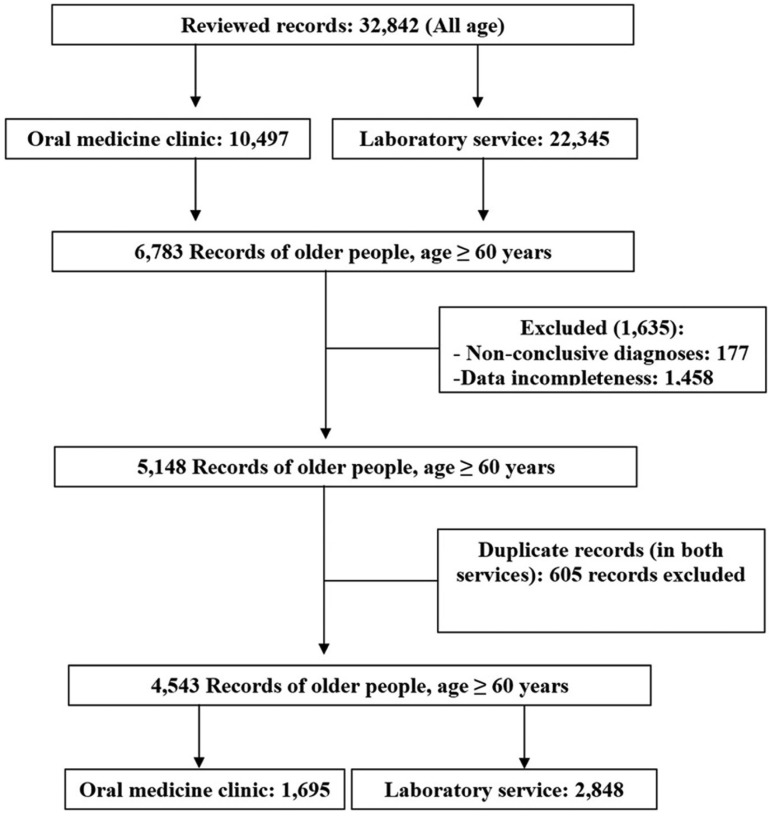
Flow diagram of the records revised in the study.

The total number of records was 32,842, of which 10,497 were from the Oral Medicine clinic service and 22,345 were from the laboratory service. A total of 6,783 (20.6%) records belonged to individuals aged 60 years or older. Among the 6,783 records, 1,635 (24.1%) were excluded from the study due to data incompleteness (1,458) and non-conclusive diagnoses (177). Of the 5,148 records of individuals aged 60 years or older, 605 records belonged to patients attending the Oral Medicine clinic, but who had also had a histopathological diagnosis. For these individuals, data from the Oral Medicine clinic were considered. Of the 4,543 records included in the study, 1,695 (37.3%) were from the Oral Medicine clinic and 2,848 (62.7%) were from the laboratory service (Fig. 1).

Missing data took place for skin color (470 cases), smoking (1,299 cases), use of alcohol (1,508 cases) and removable prosthesis wearing (2,729 cases). Demographic data of the Oral Medicine clinic and laboratory services are showed in  Table 1. Significant differences between data from the Oral Medicine clinic and data from the laboratory service were observed for sex and removable prosthesis wearing.

**Table 1:**
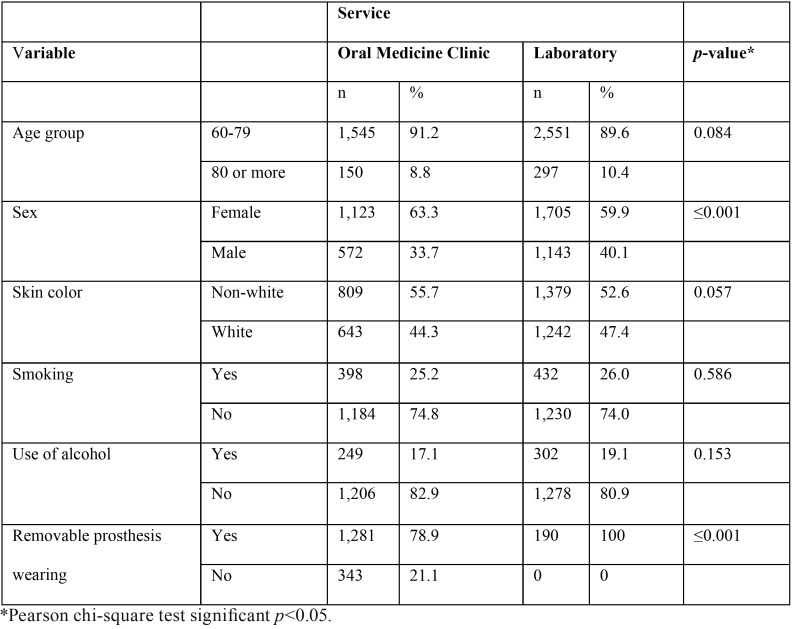
Demographic data observed in the records of the Oral Medicine Clinic and of the Laboratory Service (n=4,543).

Pain was reported in 1,202 (26.5%) cases, while in 2,468 (54.3%), lesions were asymptomatic. In 873 (19.2%) records, the symptoms were not reported. The most affected anatomical site was the alveolar mucosa (829 cases; 18.2%), followed by the buccal mucosa (576 cases; 12.7%), tongue (554 cases; 12.2%), lips (512 cases; 11.3%), and palate (504 cases; 11.1%). Other locations accounted for less than 10%.

The inflammatory/reactive lesion was the most frequent group of lesions in both Oral Medicine clinic and laboratory service ( Table 2). Differences in frequencies of the group of lesions between the Oral Medicine clinic and the laboratory service were significant for all group of lesions (*p*<0.05), except for the pigmented lesions (*p*=0.054) and for infectious diseases (*p*=0.054). Considering the lesions as a unit of analysis, the most common outcome was the inflammatory fibrous hyperplasia (IFH) in both Oral Medicine clinic (24.3%) and laboratory service (33.6%). The second and third most frequent lesions in the Oral Medicine clinic were candidiasis (15.6%) and varices (7.2%), while in the laboratorial service squamous cell carcinoma (SCC) (15.2%) and leukoplakia (or epithelial dysplasia) (12.2%) were most frequent.

**Table 2:**
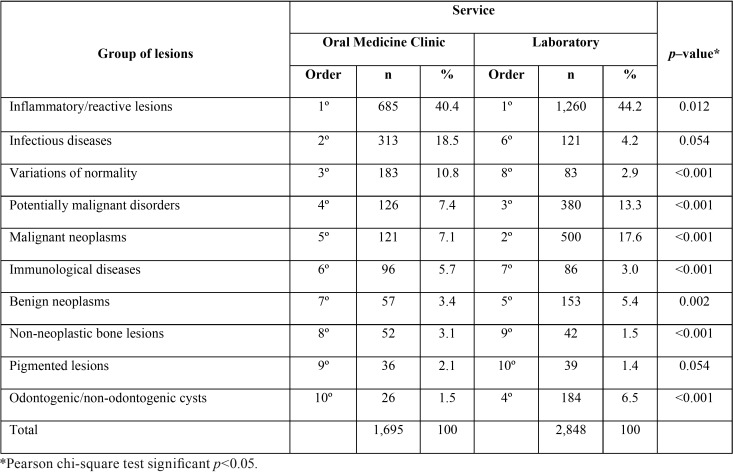
Frequencies of the oral and maxillofacial groups of lesions in older people (≥ 60 years) in the Oral Medicine Clinic and in the Laboratory Service (n=4,543).

The comparison between the young–old and old–old groups regarding the groups of lesions with data of the two services together is showed in  Table 3. The old-old individuals presented a significantly lower frequency of potentially malignant disorders (*p*=0.024) and malignant neoplasms (*p*<0.001) than the young–old group. The young–old individuals presented a significantly higher frequency of inflammatory/reactive lesions (*p*=0.011), immunological diseases (*p*=0.012) and pigmented lesions (*p*=0.013) than the old-old individuals.

**Table 3:**
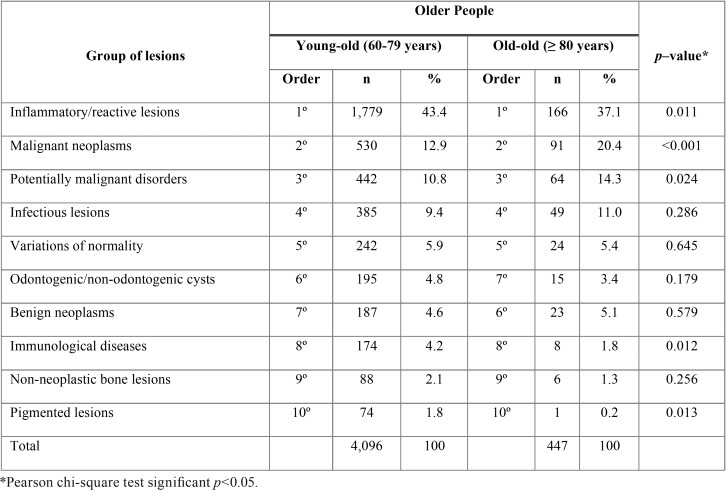
Frequencies of the oral and maxillofacial groups of lesions in each age group of older people considering data from the Oral Medicine clinic and the laboratory service (n=4,543).

**Figure 2:**
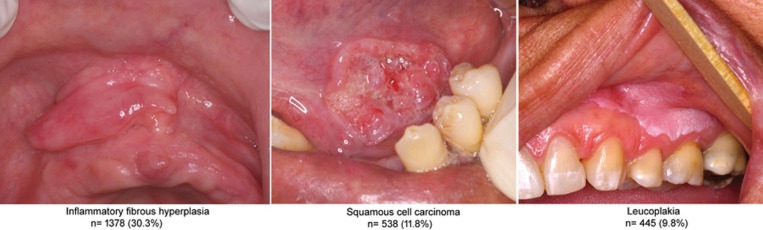
The three most frequent oral and maxillofacial lesions group in older people (≥ 60 years) in the two services together (n=4543).

The IFH (n=1,378), SCC (n=538) and leucoplakia (n=445) were the three most frequent lesions, considering the two services together (Fig. 2). The association between these lesions and the independent variables is shown in  Table 4. Young–old individuals (OR=1.33; CI=1.07–1.67), female individuals (OR=2.08; CI=1.81–2.39), and those who wore removable prosthesis (OR=3.99; CI=2.83–5.62) were more likely to present IFH than old–old individuals, male individuals, and those who did not wear removable prosthesis. Old–old individuals (OR=1.70; CI=1.30–2.21), male individuals (OR=3.63; CI=3.00–4.39), and individuals who had reported smoking (OR=6.05; CI=4.84–7.56) or alcohol use (OR=3.95; CI=3.12–5.01) were more likely to present SCC than young–old individuals, female individuals, and individuals who had not reported smoking or alcohol use. Male individuals (OR=1.51; CI=1.23–1.83) and individuals who had reported smoking (OR=2.08; CI=1.64–2.65) were more likely to present leucoplakia than female individuals and those who had not reported smoking.

**Table 4:**
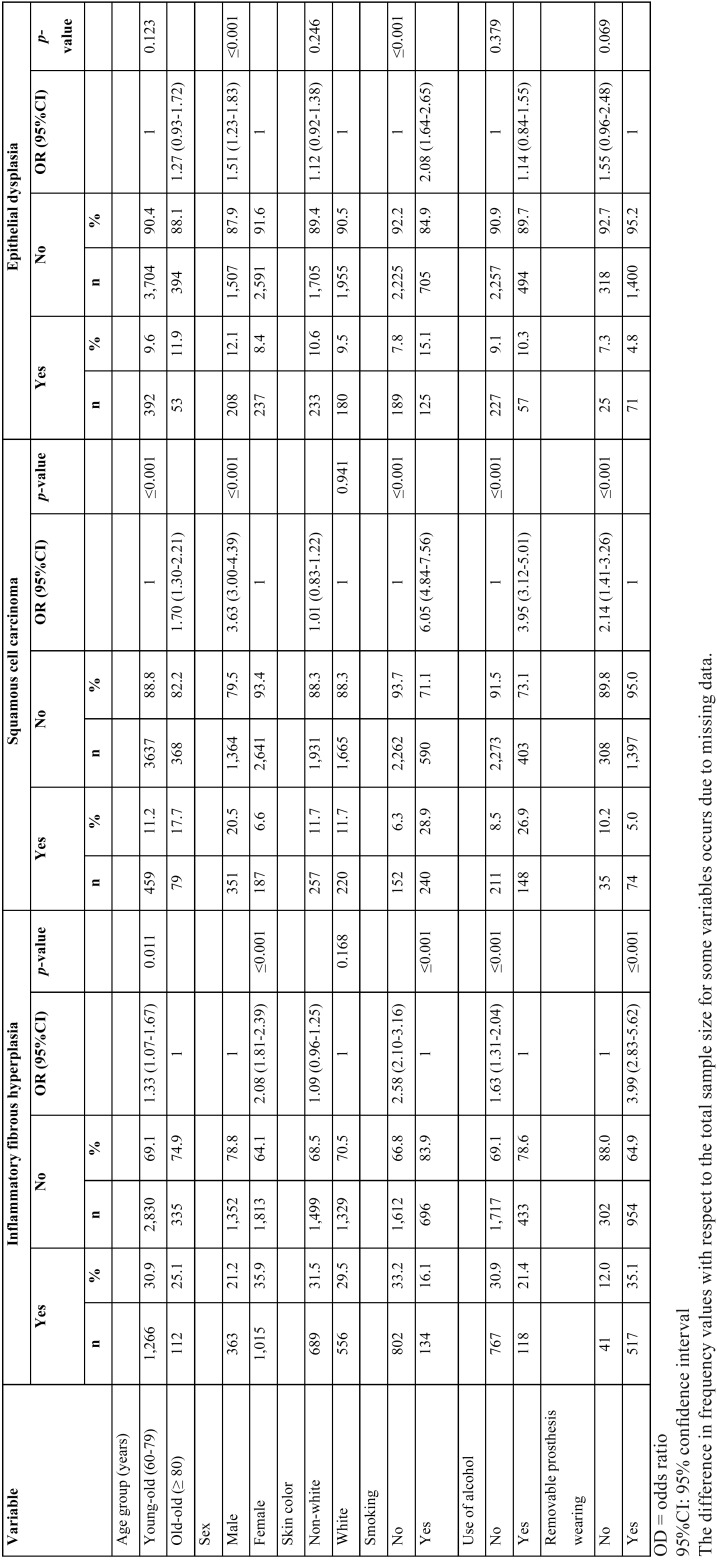
Univariate analyses of the three most frequent lesions with respect to independent variables in older people (≥ 60 years) in the Oral Medicine clinic and in the laboratory service.

## Discussion

This study evaluated the frequency of OMLs among older individuals in two different services. Our hypothesis that there was a difference in the frequency of OMLs between data from both services was confirmed. A higher frequency of infectious diseases and variations of normality across data from the Oral Medicine clinic was observed. In the laboratory service, a higher frequency of malignant neoplasms and potentially malignant disorders was observed. Most previous studies that evaluated the OMLs in Brazilian older people have been based on records from laboratory services (5,11,13). Thus far, assessments of data from Oral Medicine clinic services have been poorly described in the literature. It is important to accurately estimate the frequency of these lesions among older individuals due to the increase in the number of people over 60 years of age living worldwide. Accurate information will be relevant to health care providers, who offer health services to older individuals, in particular during the diagnosis and prevention of oral and maxillofacial diseases.

Previous studies on prevalence reported that the most common OMLs were variations of normality, fissured tongue, sublingual varicosities, and Fordyce granules (8,9). Candidiasis in complete removable prosthesis wearers is also an outcome with significance prevalence (7,18). However, similar to the present study, the most common findings in other retrospective studies were IFH, SCC, and potentially malignant disorders (10,12,13). In these studies, the records had been reviewed and the term frequency of the OMLs reported seems to be more suitable. Our results showed that there were differences in the frequencies of the lesions described in the Oral Medicine clinic and in the laboratory service, which can directly affect the information available about the most frequent OMLs among older individuals.

In our study, the laboratory service accounted for a higher number of cases because biopsied specimens from the entire state of Minas Gerais have been evaluated in this service. There were fewer cases in the Oral Medicine clinic because only individuals from the metropolitan area of Belo Horizonte have attended this service. In addition, the final diagnosis of most OMLs required histopathological exams. Of the total records in the laboratory service, approximately 15.4% were records belonging to individuals over the age of 60 years, agreeing with the findings observed elsewhere (5,10,13). A study conducted in India demonstrated a higher percentage (24%) of records belonging to older individuals. However, in this study, individuals over 50 years of age were evaluated as old people (11).

OMLs are frequently observed in older female individuals (12,13,19). Our results showed a higher percentage of women in the Oral Medicine clinic than in the laboratory service. This finding may be related to social and cultural issues. Women are more likely to seek health care than men are. Moreover, in Brazil, women have a higher life expectancy than men (20). Mandali *et al.* (21) suggested that there was an association between the frequency of lesions and the wearing of removable prosthesis for a long period due to aesthetic purposes. Coelho *et al.* (18) suggested an association between lesions in women and hormonal factors. Other studies, however, have observed that older men have a higher frequency of OMLs than women (5,6,11). Most participants, in the present study, were between 60 and 79 years old. Previous retrospective studies on data from laboratory services also had a high number of young–old individuals in their samples (12,19). Old-old people presented a higher percentage of potentially malignant disorders, benign and malignant neoplasms. There was a significant difference in the OMLs affecting individuals in each age group, which is an important issue because of the group’s vulnerability to diseases. The human aging process may cause homeostatic imbalance and may influence cellular, tissue, organic, and systemic responses, which make individuals less able to adapt to environmental stimuli, increasing their vulnerability to certain diseases. However, there is still no consensus on the physiological and pathological changes that take place among older people to confirm this hypothesis. These changes might be inherent to the process of aging or might be a response to diseases, medications, or environmental issues (3,22).

There are several different classifications that have been extensively used for the evaluation of OMLs (10,11), including the WHO guide, the International Classification of Diseases (ICD), and Oral and Maxillofacial Pathology textbooks (5,11,13,23,24). Although the group to which a lesion is assigned is determined by the nature of the lesion, discrepancies have occurred in different studies. For instance, studies have assigned lichen planus and lichenoid reaction to the group of potentially malignant disorders (11), or to the group of immunological diseases (10,12). Based on recent assessments (13,17), our study used a list of ten groups of lesions. Clear definition of a classification system is encouraged to promote uniformity and to improve communication among oral and maxillofacial pathologists.

Agreeing with results published elsewhere (10,13,19,25), in both services, the most frequent group of lesions was the inflammatory/reactive lesions. This finding may be associated with the wearing of removable prostheses by older people and may explain why the alveolar mucosa was the most affected site in this sample. IFH was less frequent among old–old individuals, as reported previously by Coelho *et al.* (18) This result may be related to the fact that old–old individuals stop wearing poorly adapted removable prostheses (26). IFH is usually caused by chronic trauma on the oral mucosa tissue of individuals who wear removable prostheses (8). The quality of the removable prosthesis, anatomical factors, and the length of time of removable prosthesis wearing may provoke the appearance of lesions. In this regard, health care deliverers should provide adequate counseling to individuals who wear removable prostheses (8,21).

Cases of SCC increased with age, as old–old individuals are more likely to present this lesion than young–old individuals. Similar results were reported by Muzyka *et al.* (27) who observed 18.6% of individuals over 85 years and 11.7% of individuals between 65 and 84 years with neoplastic lesions (epithelial dysplasia and SCC). This difference was statistically significant. In our results, male individuals and those who had reported smoking and alcohol use were more likely to present SCC, which is in agreement with the results of previous studies (8,19,28).

The third most common group of lesions among older individuals was potentially malignant disorders. Although all cases of leukoplakia were biopsied, there was a difference in the nomenclature used in each service. In the Oral Medicine clinic, the term leukoplakia was used, while in the laboratory service the term epithelial dysplasia was used. There was a higher frequency of leucoplakia observed in the laboratory service, while only a few cases of leukoplakia were seen in the Oral Medicine clinic. Old–old men and smokers were more likely to develop leukoplakia. Although there is no histological criterion to classify leukoplakia as a potential malignancy, some authors stated that lesions presenting a degree of leukoplakia and associated with risk factors, such as smoking, are more likely to become malignant lesions (29,30).

Based on the findings of this study, there is a difference in the frequency and the type of oral lesions most commonly observed among older people. Textbooks tend to report data on the frequency of lesions using information mainly extracted from studies based on biopsy sources, while data from Oral Medicine clinic services have been poorly reported. Therefore, this study adds novel information on the frequencies of OMLs, considering data from both the laboratory service (biopsies) and the Oral Medicine clinic. In clinical routine, practitioners may be more prepared to find the most common oral lesions in older people, such as infectious lesions and variations of normality. Authors and readers of books and scientific articles may be more attentive when providing statements on the frequency of lesions among the elderly, considering that the data source may have an impact on the results. In addition, the findings presented herein provide valuable information to decision makers for the distribution of financial resources and actions to meet the real demands of this population, such as campaigns for the prevention of potentially malignant disorders and malignant neoplasms in the old-old group.

The present retrospective analysis has limitations that should be recognized. First, the study’s setting was restricted to a single center. The second limitation was the study’s design, not allowing us to determine the prevalence, incidence, or any causality between OMLs and demographic and oral behavior factors. However, retrospective studies in Oral and Maxillofacial Pathology are important sources of information. They are useful to practitioners and decision makers in the public health sector, outlining what the health care provider may expect to find among old individuals with respect to OMLs and the initiatives that should be taken for the prevention, management, and control of oral and maxillofacial diseases.

The characteristics of the main OMLs observed among older people and the associated factors found in this study may be useful to practitioners and decision makers. A significant difference was observed between the age groups (young-old and old-old individuals) with respect to the groups of lesions. As regards differences between the services, inflammatory/reactive lesions and infectious diseases were more frequent in the Oral Medicine clinic, while malignant neoplasms and potentially malignant disorders were more frequent in the laboratory service. This study showed that the data source used has an impact on the results of a retrospective study assessing the frequency of OMLs among older people. Therefore, it is necessary to highlight the data source evaluated when determining the most frequent lesions among older individuals.
